# Open government data use: The Brazilian states and federal district cases

**DOI:** 10.1371/journal.pone.0298157

**Published:** 2024-03-05

**Authors:** Ilka Kawashita, Ana Alice Baptista, Delfina Soares, Morgana Andrade

**Affiliations:** 1 College of Business and Information Technology, University of Phoenix, Phoenix, Arizona, United States of America; 2 Department of Information Systems, Algoritmi Center, University of Minho, Guimarães, Portugal; 3 Operating Unit on Policy-Driven Electronic Governance (UNU-EGOV), United Nations University, Guimarães, Portugal; Public Library of Science, UNITED KINGDOM

## Abstract

**Purpose:**

This paper presents the results of an online survey and subsequent interviews investigating whether, how, and why public administrations of Brazilian states and the federal district (Federation Units) use open government data. According to the literature reviewed, the questions were categorized into four big groups: benefits, barriers, enablers, and drivers.

**Design/Methodology/Approach:**

The Survey method, based on a questionnaire followed by interviews, was used to collect and analyze data from the open data officers of 26 Brazilian Federation Units.

**Findings:**

The use of open government data is controversial as responses from the questionnaires and interviews do not match and raise questions about how well-represented each Federation Unit was. Evidence of open government data use was found. Among others, findings showed that political leadership committed to using open data facilitates and motivates public agents to use these data. Additionally, interviews indicated that the lack of human resources with the knowledge, skills, and capabilities to use open data is a relevant barrier to data use. Findings also revealed that open government data mainly support policy and decision-making processes.

**Practical implications:**

This research contributed to the open data and public administration fields. It portrays diverse realities of open government data use and institutionalization in Brazilian state and district public administrations. In addition, it provides lists of open government data use benefits, barriers, drivers, and enablers from the perspective of these administrations so that they can benchmark against each other and improve their OGD use.

**Originality and research implications:**

For academia, this research provides empirical evidence of the factors influencing public administrations’ use of open government data at the subnational level in Brazil. Even though Brazil ranks high on OGD global assessments, few studies on its use and reuse in the public sector were identified. This is one of the first academic studies focusing on open government data use in the country. It also contributes by offering to the academic community two instruments, a questionnaire and an interview protocol, which can be applied to other public settings to expand this study’s results or open new research paths by applying them to other contexts.

## Introduction

Governments worldwide are increasingly fostering a data-driven public sector as public institutions acknowledge data as an integral asset for public innovation, policymaking, service delivery, and organizational management. As a result, data use/reuse is an immense resource for the public sector [1]. Open government data (OGD) is crucial to governmental digital transformation. With OGD, public institutions make governmental data available so anyone can access, use, modify, and share it for any purpose [2].

Scholars have examined the OGD use through benefits, barriers, enablers, and drivers [3–6]. These factors can actually or potentially hinder, motivate, propel, or facilitate OGD use by the public sector. The literature recognizes that the use of these data brings benefits such as increased transparency [3,7], the development of improved public policies [4,8], and improved internal operations by creating new and improving processes, products, and services [6,9] to offer better and improved versions [10–12], and support the decision-making process [5,13].

From another perspective, the literature indicates that barriers to OGD use are the lack of organizational culture favorable to open data [3,5,11], the lack of personnel with the knowledge, skills, and/or capabilities to use open data [14–16], and low data usability [17–21].

Additionally, in order to successfully use open government data, a set of enabling factors needs to be in place. For instance, researchers identified factors like cooperative work culture [6] and government promotion of open data use [10,22] to enable or facilitate OGD use in the public sector. However, enablers alone are not enough for public sector innovation. Drivers are factors that push public agents toward using OGD [7]. Pressure from external stakeholders to use open data [23,24] and the institutionalization of open data use in the Administration [25] are drivers distilled from the literature.

According to the E-Government Survey 2022 [26], published by the United Nations (UN), the Brazilian federal government ranks 14th among 193 countries with the best digital service delivery, which is represented by the Online Services Index (OSI). Brazil ranks 49 with a very high United Nations (UN) E-Government Development Index (EGDI). According to the same publication, Brazil ranks 25th in the Open Government Data Index (OGDI). As of September 2023 [27], the Brazilian national open data portal, "dados.gov.br," published 12.186 open datasets. Researchers [28–30] have explored the publication of OGD at the federal and sub-national levels. However, little is known about OGD use/reuse, particularly by public administrations. As these data are already available, it makes sense to examine whether it is used at the sub-national level, mainly because, at this level, open data capabilities are low. The extent of open data practices is usually less robust than national ones [31]. Although the high performance of the Brazilian federal government in these areas, the situation on the sub-national level is less known and demands further scrutiny. This is one of the first academic studies focusing on OGD use by the federal district and states’ public administrations.

This study examined whether, how, and why OGD is used in the Brazilian state and district public administration. It was conducted in Brazilian states and federal district public administrations through the responses of state officials and digital transformation leaders to an online survey.

Examining the use of something requires understanding whether, how, and why it is used. Thus, to understand whether OGD is used, governmental data portals, such as the data.gov.br, offer evidence of use through use cases. However, details of why and how these data are used are limited. Researchers can examine how OGD is used by looking into who provides and uses it, what data are used for, and what categories of data are used. This research studied the use by the public sector and focused the analysis on the state level. The "why" refers to OGD use benefits, enablers, drivers, and barriers, as these factors may be considered proxies to understand why OGD is used [3–6].

In this context, three questions guided this investigation work. Q1 –*Are open government data (OGD) used in the state/district public administration*? Q2 –*How is OGD used in the state/district public administration*? Q2 addressed what OGD is used for, what categories of data are used, and which institutions provide the data. Q3 –*Why does the state/district public administration use OGD*? Q3 was addressed regarding OGD use benefits, barriers, enablers, and drivers.

These questions were answered through the conduction of a survey based on a questionnaire followed by interviews. So, this paper presents the results of this survey with a focus on the use/reuse of open government data by public administrations of Brazilian states and the federal district, which compose the Brazilian Federation Units (FUs). It is a study about the public sector’s use/reuse of public data, one of the few conducted worldwide and one of the first in Brazil.

The remainder of this article is organized as follows. The next section presents the research design adopted. A summary of the questionnaire results follows. Next, the interviews’ findings are reported. After that, the findings are discussed. Finally, the last section offers conclusions, limitations of this work, and suggestions for future work.

## Research design

Brazil was chosen for some reasons. First, Brazilian federal legislation mandates that public administration publishes open data on the federal and state transparency portals. As these data are already available, it makes sense to investigate whether the Administration uses/reuses/consumes them. Also, because there are no similar studies in Brazil, it makes sense to know if the effort to publish the data benefits the Brazilian public administration in terms of use. Lastly, because of a partnership of the University of Minho (UM), the United Nations University Operating Unit on Policy-Driven Electronic Governance (UNU-EGOV) with the National Council of State Secretaries of Administration (CONSAD), the Brazilian Association of State Entities of Information Technology and Communication (ABEP-TIC), and their Digital Transformation Group of States and Federal District (GTD.GOV), which is a Brazilian national network that gathers digital transformation specialists from state and district governments. This partnership facilitated access to respondents in the public administration and allowed uniformly reaching all states.

**Fig 1:**
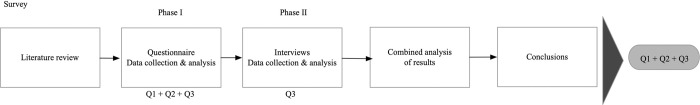
Research design.

This exploratory study examined a poorly understood field to gain insights [32,33]. The Survey research was chosen to investigate the phenomenon of OGD use by public administrations. It is a systematic research method that allows the collection of data from a representative sample of individuals to describe the practices, behaviors, thoughts, and attitudes of these individuals at a specific time and place using instruments composed of open and close-ended questions, observations, and interviews [34]. This study collected and analyzed data in two phases, as shown in Fig 1. In the first phase, an online questionnaire with 39 questions was created on the LimeSurvey platform. Responses from the questionnaires answered the questions (Q1 to Q3) that framed this work.

Ethical considerations were documented and submitted to the University of Minho’s Ethics Committee for Research in Social and Human Sciences (CEICSH), which approved the research project CEICSH 069/2021 and its Addenda 1.

After the Committee’s approval, the questionnaire in Portuguese was pretested by five people working with OGD (faculty members in higher education and master-level students in Brazil). Resulting in questions improvement for language use and content, and the logic flow was altered based on pretest results and suggestions. The English version was altered accordingly.

A non-probabilistic sampling method was adopted, as the GTD.GOV indicated 49 digital transformation leaders and managers working in the 27 Federation Units’ public to participate in the study. The potential participants received the questionnaire link by email. Participants were asked to read and acknowledge the respondent’s informed consent statement before answering the survey. The questionnaire was applied between Jun 10 and Jul 9, 2021. Questionnaire responses were analyzed.

The questionnaire results did not thoroughly address some points, so a second data collection phase, based on interviews, was designed. An interview protocol derived from questionnaire findings was developed. The interview protocol in Portuguese was pretested on two professors in higher education after the Ethics Committee approved the project addenda. The pretest was conducted in Brazil to account for the Portuguese language variations between Europe and Brazil and increase confidence in the pretest results. Feedback resulted in the exclusion of several questions to account for time constraints and clarity. All questionnaire respondents were invited by email to participate in interviews conducted in May 2022. Respondents from three states accepted the invitation.

This study builds on and expands on previous works. Categories of data used, OGD uses, benefits, barriers, enablers, and drivers were distilled from our literature review reported in [35]. Moreover, the questionnaire’s complete results were published in [36]. It takes the results of these previous works, expands them with insights from the interviews conducted, and builds new knowledge based on all these sources.

The questionnaire collected 61 responses. Thirty responses were incomplete and were discarded. Thirty-one responses remained. This study focused on the Federation Unit perspective; only the responses of the state secretaries responsible for open data policy or implementation representing the FU were of interest. As such, respondents were asked to answer from the FU perspective, not from their standpoints. This research collected data from various departments and state agencies within the same FU. Therefore, the following criteria were applied to retain a response:

- The focal points of GTD.GOV are usually allocated to the administration or planning secretariats. Thus, these secretariats’ records were selected for FUs with multiple responses.
- In addition, in the case of more than one complete response per respondent, only the last record (the one with a recent date in the last action date field) was retained.

The initial intention was to address the whole population, as representatives of all 27 FUs were invited to participate. However, São Paulo did not answer the questionnaire. Therefore, this study analyzed a non-probabilistic sample of 26 responses, one per Federation Unit representing each participating FU’s view.

Responses were exported and consolidated in an Excel spreadsheet in a tabular format. All data were then processed and cleaned, and personal data were anonymized. The last step in data preparation was translating the data from Portuguese (the language in which data were collected) to English.

Processed data were imported to Statistical Package for the Social Sciences (SPSS) and Excel for comma-separated values (CSV) format analysis. Reliability tests were conducted based on the factors or dimensions of the instrument using the results obtained from Exploratory Factor Analysis (EFA) computed on SPSS. For the state/district public administration sample (n = 16), the calculated Cronbach alphas are OGD use barriers 0.933 (33 items), benefits 0.941 (23 items), enablers 0.967 (25 items), and drivers 0.732 (6 items). The values are interpreted based on [37]. This author considers a minimum alpha of 0.7 acceptable for a reliable instrument. Thus, all calculated alphas are acceptable.

The resulting dataset was published as open data and is available as CSV files [38]. The dataset comprises five CSV files and one Excel (XLST) file. The data descriptor paper [39] comprehensively depicts the questionnaire creation and application, data collected, and associated metadata.

The second data collection phase was performed to expand our understanding of some questionnaire aspects and assemble a richer view of the phenomenon. Semi-structured qualitative research interviews were conducted to collect facts and experiences about OGD use benefits, barriers, drivers, and enablers in Brazilian public administrations. This phase was conceived to address only Q3 because the questionnaire responses did not thoroughly clarify some factors. However, considerations about Q1 and Q2 emerged during the interviews’ interactions, as knowledge was collaboratively constructed.

The interview questions were selected based on questionnaire responses that diverged from the literature findings. For example, the benefit BEE03—*Reduced operating costs* is considered relevant in the literature [40,41]. However, 13% (2) of the questionnaire respondents reported this benefit as irrelevant. Other questions selected obtained high rates of disagreement and neutrals. For instance, BAPS02—*Strategy and/or leadership do not support open data use* got 50% (8) of neutral and 31% (5) of disagreement.

All questionnaire respondents were invited for interviews by email. Representatives of three states accepted to participate. Two individual and one group interviews were conducted, as two interviewees represented one state. Interviews were performed in Portuguese and were automatically transcribed using the NVivo transcription software. A human examined and further transformed the transcriptions to improve understandability and readability. To anonymize interviewees, they were named ENT4, ENT5, ENT6, and ENT7. Then, transcriptions were automatically translated into English using Google and Microsoft translators. A bilingual researcher further adjusted translations. Interviews were conducted on the Zoom platform. Qualitative data analysis was executed using the NVivo platform. Initial manual coding was seeded with existing questionnaire factors, and new ones were added as they emerged during analysis.

The extracts of transcriptions and corresponding translations used in this article and other reports related to this investigation were published as open data [38].

Personal data were collected (IP address, emails, gender, and age), and they were anonymized to prevent individual respondent identification. An Informed Consent Form was sent by email to interviewees, who later verbally consented to participate. Interviews were recorded and will be destroyed after the reporting phase. Partial transcripts were produced and anonymized.

## Results and discussion

In this section, questions Q1, Q2, and Q3 that framed this study are answered based on the questionnaire results and the narratives of the three interviews. These narratives were examined and provided evidence that corroborated or contradicted the questionnaire results.

### Q1 results and discussion

Q1 –Are open government data (OGD) used in the state/district public administration?

**Fig 2:**
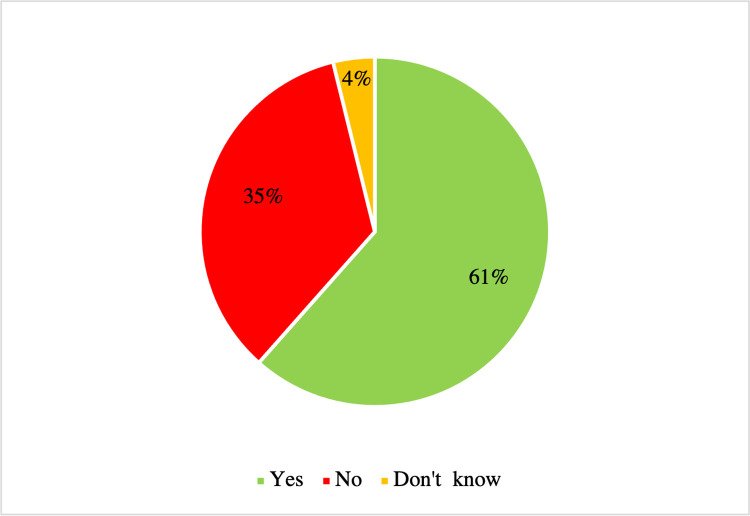
Are open government data used in state/district administration? (n = 26).

As illustrated in Fig 2, questionnaire results show that 16 (61%) of the Federation Units use OGD, nine (35%) do not use it, and one (4%) does not know if OGD is used.

Two of the interviewed states reported using OGD in the questionnaire. However, interviews provided evidence that only one of these states uses open data, as ENT5 reported, "For the early childhood program, we managed to dimension the demand for two hundred daycare centers […] their locations too. All this was based on available data, and some of them are open data."

The use of open data was not mentioned during the interview with the second state. The third state interviewed reported in the questionnaire not knowing if open data were used. However, the same interviewee reported using open data in a previous position in the same state, “[…]in my regular job of forecasting revenue and expense, when an increase that had been granted for a specific career, [I] needed information on the transparency portal…."

Although the interview indicated that open data is used in the state, its use is not institutionalized. In addition, the state official responsible for implementing the open data policy reported being concerned with raising open data awareness and related that he seeks specific projects with areas that could have a demand and invites them to implement it using [open data].

This intention is aligned with current literature [1], which recommends that agencies proactively encourage the use/reuse of this data outside and inside the public sector through data awareness projects, hackathons, co-creation events, information sessions, and regular training for public servants. In addition, other researchers suggest that agencies publicize their open datasets through social media and their open data portals to raise engagement and awareness of their open datasets [42].

### Q2 results and discussion

Q2 –How is OGD used in the state/district public administration? Q2 addressed what OGD is used for, what categories of data are used, and which institutions provide the data.

**Fig 3:**
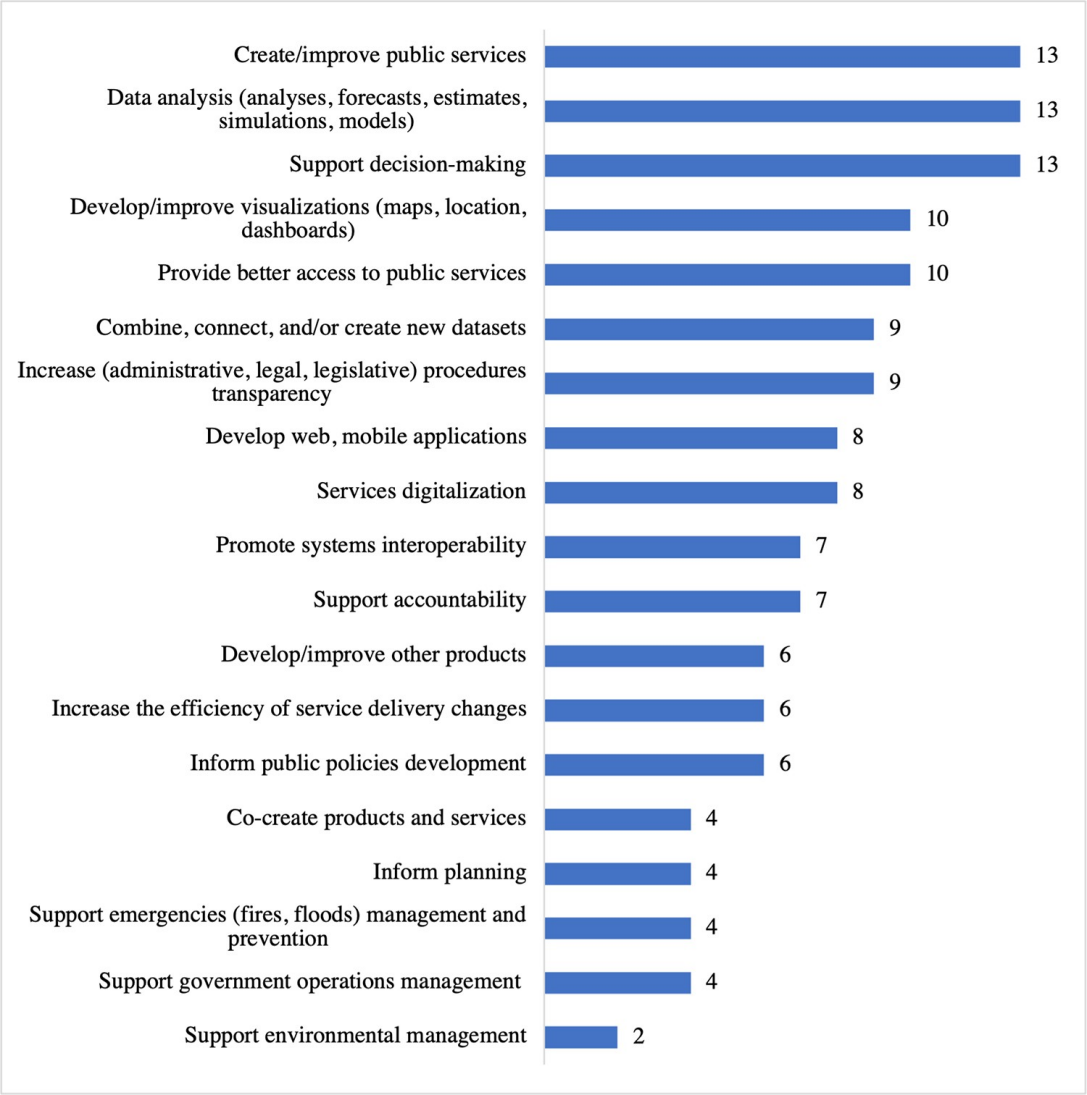
What open government data are used for (n = 16).

As presented in Fig 3, responses indicated that OGD is primarily used to support decision-making, create/improve public services, and analyze data. ENT4’s narrative in the Q1 discussion supports the questionnaire results in that data is used to create forecasts. Additionally, ENT5 discourse adds the formulation of data-driven public policies as another OGD use, "The government uses open data to calculate socioeconomic indicators, for example, gross domestic product, to formulate data-driven public policies…."

Interviewees claimed OGD could improve the decision-making process and access to public services, increase administrative efficiency, improve public policies, develop new processes, products, and services, and improve governance. ENT5 offered insights into some of these uses with the Early Childhood program partially based on open data and related, "It was possible to formulate [public policy] because data was available… ."

These findings support previous research on the improvement of the decision-making process [5,9], the improvement of access to public services [43–45], and the development of new processes, products, and services [3,9,46]. These results seem reasonable for the public sector as governments strive to transform operations digitally. Governments need to understand how to make meaningful decisions for digital government in the domestic context [47], so successful digital transformation will allow the public sector to deliver public services that are simpler and more effective and to operate efficiently and effectively in the digital environment [48].

The three most popular OGD categories mentioned by 10 (63%) respondents were procurement, expenditure, and budget. The three most-cited data providers were the State Secretariats, the Brazilian Institute of Geography and Statistics (IBGE), and the Comptroller General of the State (CGE).

### Q3 results and discussion

Q3 –Why does the state/district public administration use OGD? Q3 was developed regarding OGD use benefits, barriers, enablers, and drivers. These factors may be considered proxies to understand why OGD is used. Therefore, results are reported according to these factors.

### OGD use benefits

**Fig 4:**
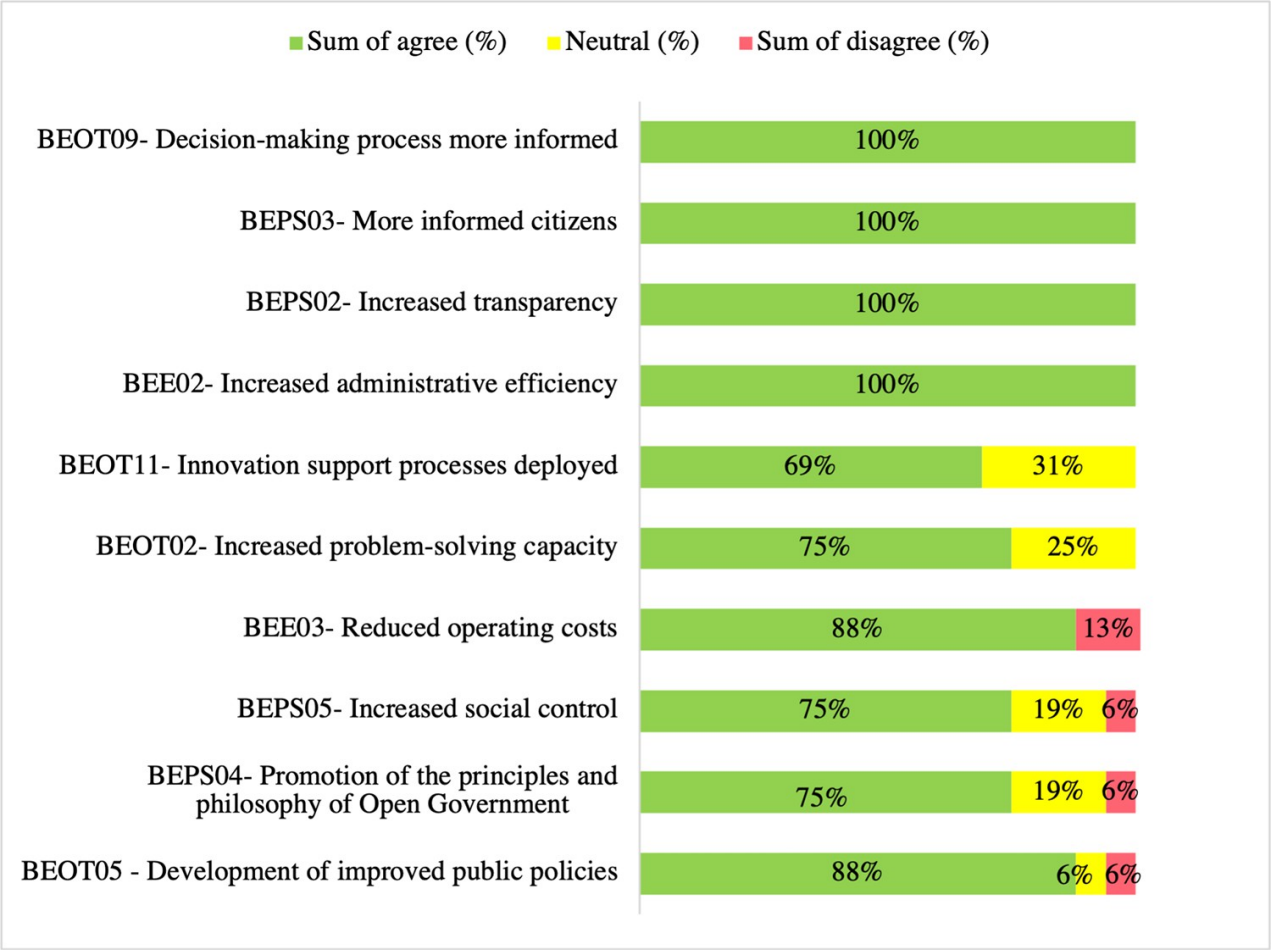
OGD use benefits questionnaire responses ranked topmost agree, neutral, and disagree (%) (n = 16).

As seen in Fig 4, respondents agreed with the benefits identified in the literature since none got less than 69% (11) of agreement (the simple sum of strongly agree and agree). Moreover, they did not strongly disagree with any benefit surveyed. However, they disagreed with BEE03—*Reduced operating costs*, BEPS04—*Promotion of the principles and philosophy of Open Government*, BEPS05—*Increased social control*, and BEOT05—*Development of improved public policies*. Thirty-one percent (5) of respondents demonstrated neutrality towards BEOT11—*Innovation support processes deployed* being a benefit. Moreover, 25% (4) of respondents also reported neutrality towards BEPS06—*Increased civic participation and public engagement* and BEOT02—*Increased problem-solving capacity*. The respondents agreed that BEE02—*Increased administrative efficiency*, BEPS02—*Increased transparency*, BEPS03—*More informed citizens*, and BEOT09 –*Decision-making process more informed* are the most relevant OGD use benefits. Previous research supports these results, recognizing BEPS02—*Increased transparency* [4,6,49,50] and BEOT09 –*Decision-making process more informed* [4,13,51–54] as relevant OGD use benefits. These findings make sense, as the public sector needs reliable data to make decisions to improve the transparency of its acts and increase administrative efficiency. Despite receiving a 76% (12) agreement rate in the questionnaire and being a well-acknowledged benefit in the literature [6,55,56], BEPS06—*Increased civic participation and public engagement* obtained a 25% (4) neutrality response rate.

Interviewees indicated that BEE03 –*Reduced operating costs* is a potential benefit not yet realized. When asked about it, ENT05 commented that this benefit is more of an expectation than a reality [. ENT7 offered a divergent view,

"If I have these [data] well-structured, well organized, and classified when needed, you know where to look for it. So, it will generate a huge reduction, and we have duplicated data in four state databases. There would be no need for that."

These contradictory findings raise questions when analyzed in light of the literature [4,40,41,43] that considered it a use benefit, as reusing these data reduces the cost to find and make it available each time it is needed. So, why did it obtain a 13% (2) sum of disagreement in the Brazilian state and district public administration context? One explanation could be that it is difficult to calculate these initiatives’ savings, given the high investments to create and maintain the technology to support open data.

In the questionnaire responses, BEOT11 –*Innovation support processes deployed* got the topmost neutral rate (31% (5)). However, the interviewee’s narrations signaled it as a potential benefit not yet realized in this context. For example, ENT05 commented, "… a process that supports innovation implemented, I would say that open data has this potential, but it is not automatic. It is necessary to have managers who understand open data potential to promote innovation." ENT6 offered, "The open data process is one of the biggest input axes for innovation. Intelligent government with governance, e-government, everything is based on open data. Additionally, an intelligent government uses open data for co-production with the citizen, the user …." The literature [5,40] agrees with this view.

In short, respondents indicated that OGD use has the potential to reduce operational costs and support the deployment of innovation processes. However, these benefits were not yet realized in this setting.

The interviewees’ discourses identified the following benefits: BEOT09—*Decision-making process more informed*, BEOT5—*Development of improved public policies*, and BEOT01*- Improved access to public services*. ENT5 reported that the availability and access to OGD allowed the formulation of a policy that generated and provided almost 900,000 people with the opportunity to complete their elementary education.

Other extracts were gathered during the interviews and illustrated additional reasons why OGD is used, expressed in terms of OGD use benefits. For instance, BEE–2—*Increased administrative efficiency*; ENT4 stated,

"The Open Data Portal brings this efficiency gain for me and other public service users. Internally, within the state, there is much difficulty in disclosing information that should already be accessible […] with the same requirements that apply to open data."

From the questionnaire responses, the most prominent OGD use benefits reported are increased administrative efficiency, increased transparency, citizens being more informed, and more informed decision-making. Interviews provided evidence of these and other benefits mentioned by interviewees, such as ENT5 and ENT7.

These results are consistent with previous research that indicated OGD use increases administrative effectiveness and efficiency by implementing evidence-based decision-making processes, reducing work duplication within the government, and improving the access and provision of public services [40,43,45]. Moreover, governments implement OGD initiatives to improve transparency and provide better services for society [5,9,41].

The following section presents the results and discussion regarding OGD use barriers.

### OGD use barriers

**Fig 5:**
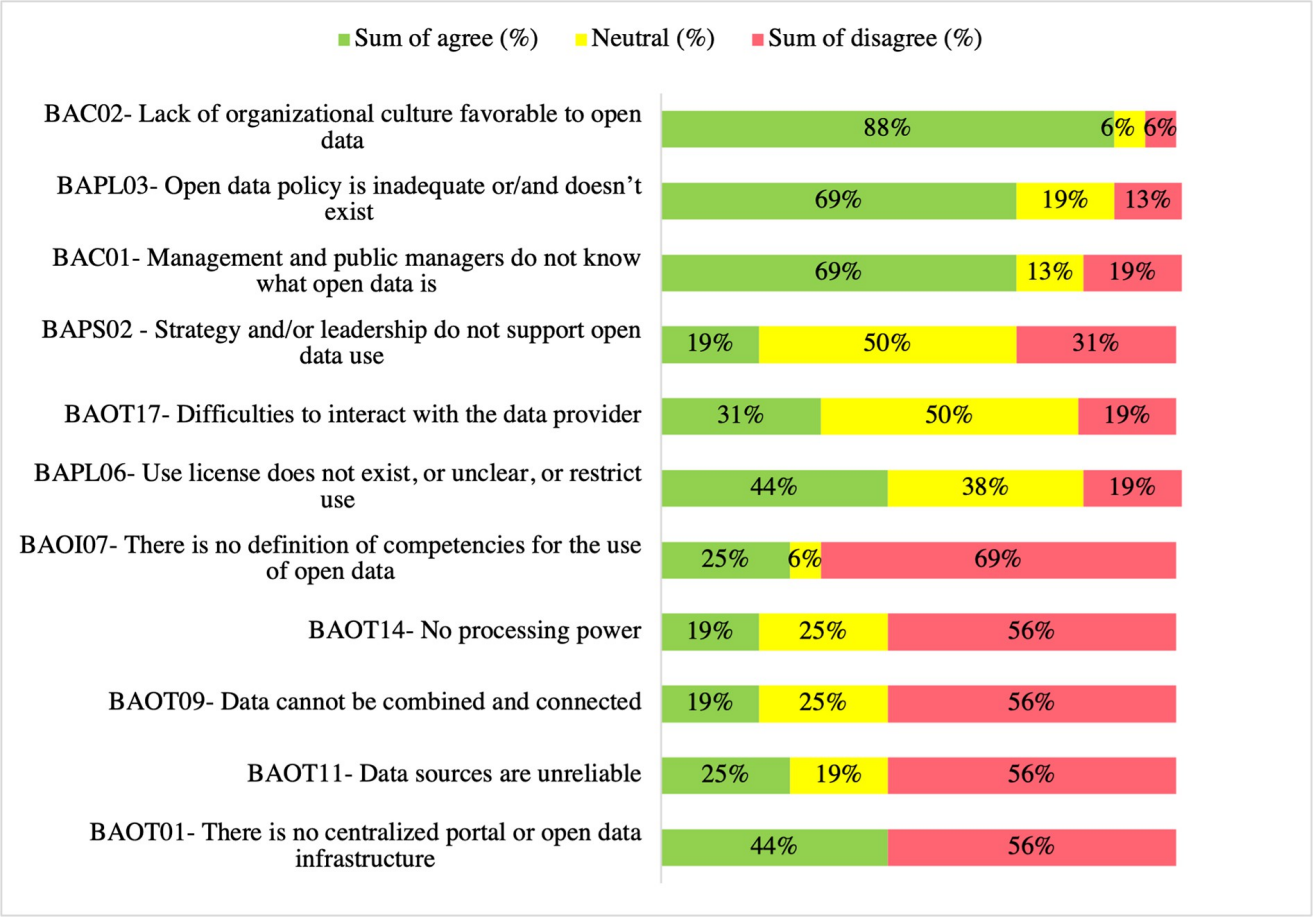
OGD use barriers questionnaire responses ranked topmost agree, neutral, and disagree (%) (n = 16).

Fig 5 shows the highest sum of agrees, neutral, and the sum of disagrees results for the question—What are the barriers to using open government data? The barriers that obtained the highest sum of agreements are BAC2—*Lack of organizational culture favorable to open data* (88% (14)) and tied with 69% (11) are BAC1—*Management and public managers do not know what open data is*, and BAPL3—*Open data policy is inadequate or/and does not exist*. Two barriers obtained a 50% (8) neutrality rate: BAPS2—*Strategy and/or leadership do not support open data use* and BAOT7—*Difficulties to interact with the data provider*. These barriers were candidates for a closer examination in future work because of their neutrality rates. BAOI7—*There is no definition of competencies for the use of open* data received the highest sum of disagree rate, 69% (11). This finding is unsurprising given the public sector context, as public entities and agents are bound to do what the law prescribes. So, a set of competencies must be defined before a public entity starts publishing and using open data. Moreover, it does agree with the literature [11,57] that reported that this lack of definition of competencies negatively affects OGD use.

The barrier BAPL06—*Use license does not exist*, *or unclear*, *or restrict use* was among the three top tiers of neutral (38% (6)) and got a disagree rate of 19% (3) in the questionnaire responses. When asked whether BAPL06 was relevant in the context of the FU, ENT5 commented,

"This theme of the use license is an advanced theme. It is important [….]The vast and gigantic majority of users have no notion that this exists. Additionally, they are not impacted by it by not having any notion that this exists.”

Even though the FU lacks an open data policy, ENT7 indicated being aware of the issue from the publisher’s perspective, “When you enter this part of license use, the indefinite part, and restriction, as we do not have an adequate policy, we do not know what can be shared or not, what is classified as confidential or not, is binding or not.”

Although interviewees acknowledged its relevance, narrations suggested that the barrier BAPL06—*Use license does not exist or is unclear restrict use* was not considered relevant because many users are unaware of this issue. Questionnaire results reported that 47% (9) of the respondents were neutral or disagreed that the lack of use license was a significant barrier. However, these findings disagree with some researchers [9,22,58,59], who considered the absence of open license information a critical barrier to the effective use of OGD, as the license provides the legal assurance that the user risks no penalties for using it.

Interviewees were asked to comment on the significance of BAPL05—*Standards for using non-existent and/or inappropriate open data*. ENT5 offered,"… it is not a barrier to open data use because regardless of standards, people often access data on the Internet in general and use it. The patterns increase the potential for the use of open data.”

ENT7 informed that despite these standards’ relevance, the state lacks them as it does not have an open data policy.

These narrations suggest that although relevant, the lack or the inadequacy of open data standards hinders but does not prevent OGD use. It may justify the 31% (5) neutral and 13% (2) disagree rates in the questionnaire responses. This finding accords with previous research [57,60] that considered BAPL05 a relevant barrier because the lack of these standards increases the complexity of using OGD, making it difficult to combine multiple data sets and application program interfaces (APIs).

Interviewees were invited to share their experiences about the OGD use barrier BAOT12—*Results obtained from different sources differ*. Questionnaire responses indicated 25% (4) neutral and 38% (6) disagree rates for this barrier. ENT05 agreed that it is a barrier. However, he indicated that usually, the problem is not in the data but instead in how this data is generated, especially when used for developing indicators.

BAOT10—*Data is not sustained or maintained* obtained 25% (4) agree, 25% (4) neutral, and 50% (8) disagree rates in the questionnaire responses. When asked about this barrier’s relevance, ENT5 stated,

"… this is also an important barrier because from the moment the dataset is not sustained or maintained, it comes close to being useless data except if it has historical value.” ENT7 offered, "Yes, [the barrier is relevant] because data may be outdated. In reality, data becomes outdated…."

Despite the high disagreement and significant neutral rates obtained in the questionnaire responses, the interviews’ findings agree with the literature [46,60] that indicated that users are reticent to use data that are not maintained and sustained, as there is no insurance that the data will be available in the future.

Questionnaire responses indicated mixed results—25% (4) agree, 25% (4) neutral, and 50% (8) disagree—for BAOI08—*There are no human resources with the knowledge*, *skills*, *and/or capabilities to use open data*. The questionnaire result was unexpected as it contradicts previous research [46,57,59–62], which extensively reported that public agents lack the knowledge and skills to manipulate open data, and promoting initiatives to capacitate them is critical for fostering OGD use [42,63]. However, ENT5’s narration agrees with the literature, “This is an extremely relevant barrier because if human resources don’t have the literacy to use open data […], they won’t use it, or they can even misread what the data reports.”

Regarding BAPL03—*Open data policy is inadequate or/and does not exist*, ENT4 related, "Then [open data] ends up being a new policy here in the State ZYX." ENT 6 said, "… we are starting to create [open data] policy right now, here in the state XYZ… .” ENT7 reported, "… today we will not have an open data policy.”

These narratives indicate that the lack of open data policies, standards, and guidelines is a relevant barrier to OGD use, as states that do not have this policy implemented lack the institutional support to develop the required capabilities to publish or use open data.

This finding is aligned with previous research, which reported that the lack of a well-defined open data policy and regulations [46,58,62], OGD use standards and guidelines [3,57,59], and appropriate open data and infrastructure [61,64] hinders the publication and subsequent use of these data.

The following section presents the survey results and discussion of OGD use enablers.

### OGD use enablers

**Fig 6:**
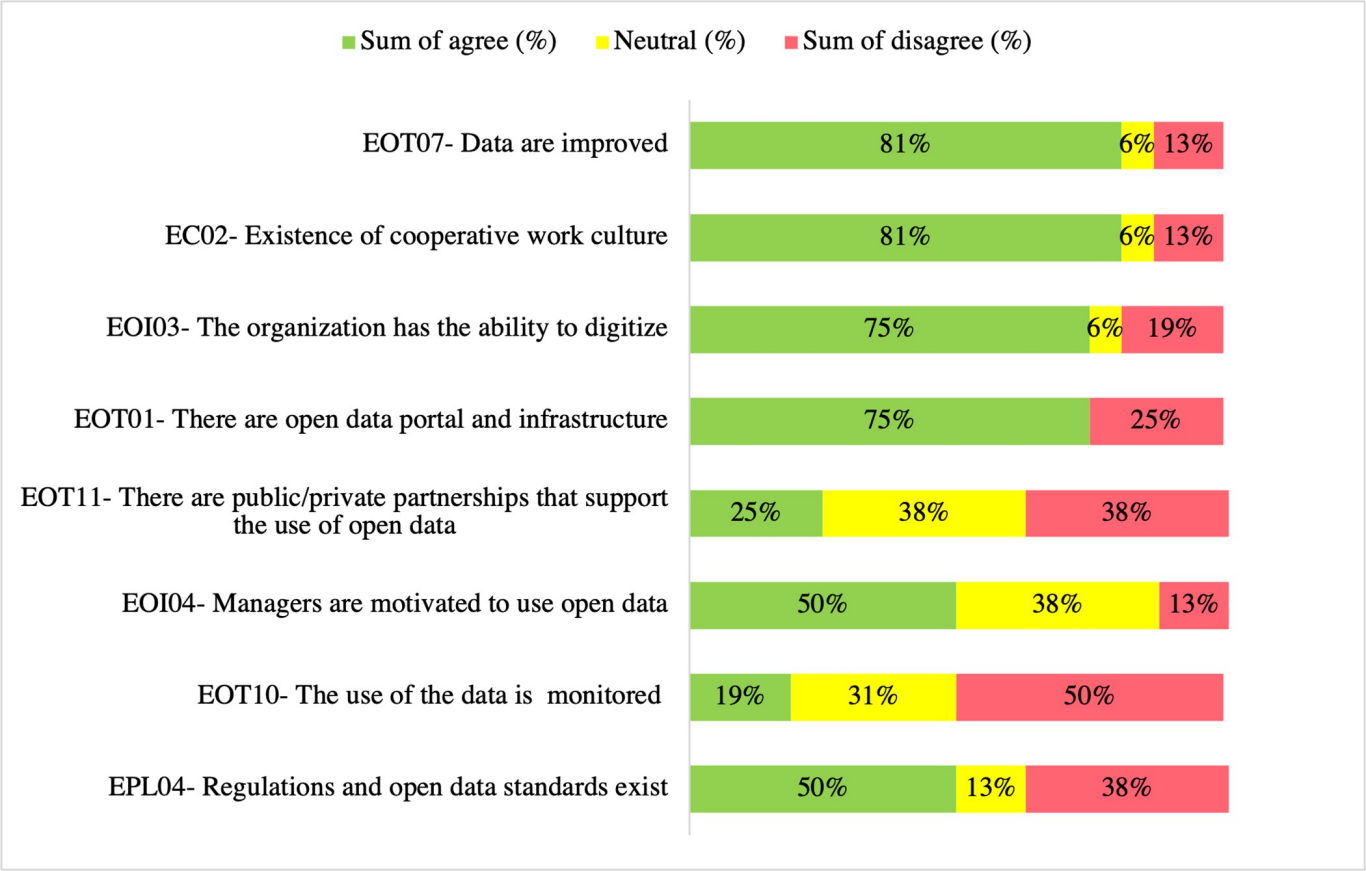
OGD use enablers questionnaire responses ranked topmost agree, neutral, and disagree (%) (n = 16).

Fig 6 shows the highest sum of agrees, neutral, and the sum of disagrees responses to the questionnaire question—What are the enablers of open government data use? The enablers with the highest sum of agree 81% (13) are EC02 –*Existence of cooperative work culture* and EOT07 –*Data are improved*. The top-rated neutrals, 38% (6), are EOT11—*There are public/private partnerships that support the use of open data* and EOI04—*Managers are motivated to use open data*. Based on their neutrality rates, they were candidates for further examination, along with EOT10—*The use of the data is monitored*, which obtained the highest disagree rate of 50% (8).

In the interviews, participants were asked about the relevance of OGD use enabler EOI04—*Managers are motivated to use open data*. The conversation brought up another enabler, EPS01—*Political leadership committed to the use of open data*, which seems to be an enabler of open data use among the ranks. ENT5 related, "It facilitates very much, […] we had a planning secretary […] who guided the team very well to build whole systematic decision-making. Not only that, but very advanced public policy formulation too, […] strongly based on data."

This narration suggested that political leadership committed to open data use motivates the tactical and operational levels to use it. These findings are supported by previous research, which signaled that political leadership’s will, direct support, or commitment is an important enabler in setting a vision and the basis for OGD implementation and use [61,65].

When asked about the relevance of EOT10—*Data usage is monitored and tracked*, ENT4 explained, "We publish the [open] data and are responsible for the portal. However, we still don’t have a clear policy of monitoring usage internally within the public administration.” ENT7 commented, “This is important. The point is that, in the state, we are not yet at this level.” Thus, these narrations indicate that these states have not yet reached a level of open data maturity to be concerned with monitoring and tracking OGD use. Questionnaire results reported that only 19% (3) of the respondents agreed, 31% (5) were neutral, and 50% (8) disagreed. These findings are not aligned with previous research [49,66,67], which stated that the ability to track and monitor OGD use provides evidence that OGD is being accessed and used, which by itself, may also provide public entities some justification for adopting transparency and open government initiatives and warranting government investments on open data technology.

Regarding EOT11—*There are public/private partnerships that support the use of open data*. ENT5 stated, “I’ve never seen such a partnership work.” ENT6 and ENT 7 reported that if there are any partnerships, they might be between the state and federal governments., These interviews suggest that public/private partnerships are uncommon in these states. While the questionnaire responses indicated mixed results, 25% (4) agreed, 38% (6) were neutral, and 38% (6) disagreed. This finding diverged from the literature [4,6,10,22,62,68], which offered partnership examples and reported they were used to foster OGD use by providing, among other resources, financial support to open data initiatives, the infrastructure and standards for OGD and data intermediaries to manipulate data and develop applications."

The following section presents the results and discussion about OGD use drivers.

### OGD use drivers

**Fig 7:**
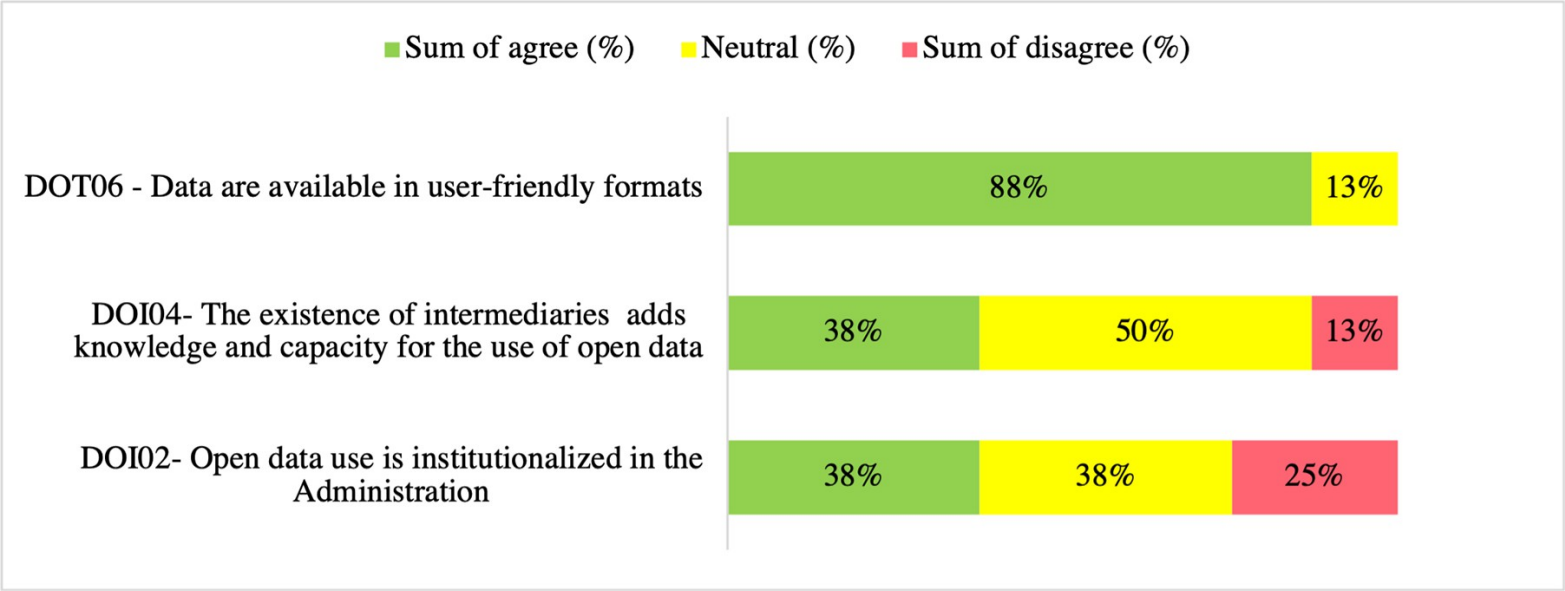
OGD use enablers questionnaire responses ranked topmost agree, neutral, and disagree (%) (n = 16).

Fig 7 presents the highest sum of agrees, neutral, and the sum of disagrees results for the questionnaire question—What are open government data use drivers? The OGD use driver with the highest sum of agrees 88% (14) is DOT06—*Data is available in user-friendly formats*. The top neutral 50% (8) is DOI04—*The existence of intermediaries adds knowledge and capacity for the use of open data*, and the highest sum of disagree 25% (4) is DOI02—*Open data use is institutionalized in the administration*. Therefore, these drivers may be more scrutinized based on their rates.

Results in the questionnaire indicated that the driver DOI04—*The existence of intermediaries adds knowledge and capacity for using open data* as the neutral top rate 50% (8), 38% (6) agree, and 13% (2) disagree. During interviews, the interviewer explained that an intermediary could be a person or institution that provides the organization with the capabilities to work with open data. After the explanation, interviewees were asked about DOI04’s relevance. ENT5 commented, "In this sense, I can answer that intermediaries have a crucial role […] of decoding these data so that users can more easily consume it."

ENT7 explained that intermediaries would be hired to do the job because the state lacks the capabilities and knowledge to work with open data.

In short, interviewees considered intermediaries as agents facilitating OGD use, not as a driving force behind the phenomenon. These findings align with researchers who regarded data intermediaries as actors that acquire, use, and transform data for the public to foster civic participation and increase transparency [9,69]. However, other scholars argued that as citizens need to acquire specific skills and tools to use these data, usually beyond their capabilities [40,70], there is a risk of relying on biased intermediaries, which could mislead the overall public interest and objective of the intentionality of opening the data [40].

In the questionnaire, respondents claimed that the top two OGD use drivers are data being available in user-friendly formats and external stakeholders (international bodies, other bodies, journalists) pressing the administration to use open data. Interviews did not address these drivers directly. However, the lack of data available in user-friendly formats surfaced as a barrier. ENT4 reported, "…[I] needed information on the transparency portal, but I could not process it because of the format that was distributed."

These findings accord with previous literature that cited low data usability and lack of metadata as barriers to OGD use [17,57,71]. Moreover, the availability of datasets in user-friendly formats with detailed metadata is considered an OGD use driver [72].

The following section concludes this paper, presents this study’s limitations and contributions, and suggests paths for future works.

## Conclusions, limitations, and future work

This study examined whether, how, and why the Brazilian state and district public administrations use OGD. Evidence of OGD use was found. Although results reported a limited view of OGD use in Brazilian states and federal district public administrations, they offer a starting point for state decision-makers, public officials, and practitioners’ action. For instance, they can use these factors to change and improve strategies, policies, and data-use processes to implement facilitating measures to surmount barriers and promote OGD use to realize positive benefits. In this context, the findings:

- Showed that political leadership committed to using open data facilitates and motivates public agents to use these data. Thus, FUs should raise political leadership, public officials, and agents’ awareness of open data by publicizing their open data initiatives, the FU data portal, and published datasets, and also by giving examples of its good use;
- Indicated that a lack of human resources with the knowledge, skills, and capabilities to use open data is a relevant barrier to OGD use. Therefore, FUs should need to offer training on open data technologies to public managers and agents;
- Revealed that the OGD is mainly used for supporting policy and decision-making processes. FUs play two independent roles: as a publisher and as a user. As the latter, FUs need data to be available, accessible, and of high quality so it can be used to support policy and decision-making processes. As a publisher, the FU ensures that published data follows open data standards, has the appropriate license, and is sustained. Thus, although these roles are independent, FUs should streamline their open data publication process so it will provide high-quality data and obtain it from other FUs. These data also can potentially be used by international governments and organizations, bringing to attention interoperability and idiom aspects;
- Indicated that the use of OGD by governments increases the efficiency of the administrative machine. It reduces and improves the quality of their expenses, benefiting society.

Limitations of this study are the sampling approach and the number of questionnaires analyzed, as the study, by design, sought one response per Federation Unit. The whole population is limited to a maximum of 27 FUs. Twenty-six (26) FUs answered, and the questionnaire analysis considered one response per participating FU. Although participants represented the FUs and were asked to answer interviews from the FU perspective, during the interviews, some passages raised doubts about whether they represented the personal view instead of the institutional viewpoint. Also, in some cases, there may be confusion between facts and opinions, making it difficult to distinguish them. Some interview narrations did not support questionnaire responses, suggesting that participants may not have in-depth knowledge of the FU-wide phenomenon. Therefore, applying the questionnaire to other secretariats could broaden and enrich our view of OGD use in these public administrations. An additional limitation is the number of interviews conducted. The 26 questionnaire respondents were invited; however, only three accepted and were interviewed. Their responses were used to complement and consolidate the questionnaire results. Interviews accounted for cases where OGD is not yet used, sparsely used, and effectively used, attesting to the diversity of contexts of the states. Performing more interviews or a panel with all states could enrich these contexts and develop new ones.

Generalization was not an objective of this research, as knowledge built during interview conversations could be transferred to other situations accounting for the context and heterogeneity of social knowledge [73]. Answers are deemed valid, as respondents were able to communicate their experiences in comprehensible and meaningful ways [74]. Interviews were conducted based on leading questions, which were used to repeatedly verify the reliability of interviewees’ answers and validate the interviewer’s interpretations. Therefore, the knowledge constructed results from collaborative and informal interaction between the interviewer and interviewees [73]. Other Federation Unit administrations can use the findings to help them pave their way to using open data. They give an account of a state that effectively uses open data to tackle social problems and could be used as a study case to foster use. Additional interviews with all the other FUs, public/government officials, and other secretaries within the FU would be necessary to broaden our understanding of OGD use in the Brazilian states and district public administrations.

Another interview limitation was that the term "intermediaries" used in the questionnaire did not have a clear meaning for interviewees. So, the results could have been slightly different if the question had been worded differently. Additionally, from the narratives, intermediaries can be seen as enablers, not drivers of OGD use.

Due to time constraints, the interviews addressed only some questions that the questionnaire responses did not fully answer. Therefore, a path for future work is to broaden the scope of interviews with other FUs.

This research contributed to the open data and public administration fields. For practitioners, it has portrayed the diverse realities of OGD use and its institutionalization in Brazilian state and district public administrations. In addition, it provides lists of OGD use benefits, barriers, drivers, and enablers from the perspective of the Brazilian Federation Units so that they can benchmark against each other. This information is helpful as it provides evidence for decision-making and policymaking regarding the OGD adoption and implementation in Brazil. Moreover, the study and its usefulness for such policymaking are relevant because it can guide decisions, regulatory documents, and policies defined even at the national level.

Finally, for academia, this research provides empirical evidence of the factors influencing the use of OGD at the subnational level in Brazil. It contributes to theory and academia with two instruments: a questionnaire [75] and an interview protocol [76]. They can be applied to other public settings, expanding this study, or to private settings, opening new research paths. The survey could be a starting point to broaden OGD use research at the institutional level. Exciting research venues envisage expanding this study by applying the survey to other Brazilian government spheres to understand OGD use differences and similarities.

Moreover, how do the Brazilian results compare with other countries? Another research path could apply the survey in other countries to obtain a picture of OGD use or to compare results. This paper also contributes to bridging the gap in works focused on the use of data and the use of data by the public sector itself. This is the space of this work, and it is that gap, which is little explored, that this investigation fills.
